# Inclusive Approaches To Promote Participation of Diverse Demographic Groups in Preclinical AD Clinical Trials Through Centralized Recruitment

**DOI:** 10.1002/alz.087612

**Published:** 2025-01-09

**Authors:** Doris P. Molina‐Henry

**Affiliations:** ^1^ University of Southern California, San Diego USA

## Abstract

**Background:**

Recruitment of demographically diverse participants into Alzheimer’s disease (AD) clinical trials, encompassing both screening and randomization, remains a consistent and persistent challenge contributing to underrepresentation of certain groups. Despite the exciting prospects of identifying therapeutic interventions for biomarker‐eligible, cognitively unimpaired individuals, these studies grapple with the inherent complexities of AD trials coupled with intricate and time‐consuming screening processes. Addressing this the issue of underrepresentation necessitates concerted and intentional efforts that prioritize inclusivity and equitable access to enroll adults meeting study criteria, reflecting the demographic and social diversity of North America. This presentation delves into the strategic approach undertaken by the Recruitment, Engagement, and Retention Unit (the Unit) within the National Institute on Aging‐funded Alzheimer’s Clinical Trials Consortium (ACTC).

**Methods:**

The Unit defines and monitors populations of emphasis across dimensions of diversity such as race, ethnicity, sexual and gender minorities (LGBTQ+), and socially minoritized and underserved groups to underscore inclusivity. Guided by principles of research access, media outreach, site engagement, and community partnerships, the Unit implements a dynamic multi‐pronged strategy across clinical trials that span several stages of the disease.

**Result:**

Research access involves evaluating and adapting messaging in digital and print formats, ensuring linguistically compatible communication, and providing informational resources tailored to these specific groups. Media efforts include earned and print media interviews across a variety of outlets focused on engaging communities of emphasis. ACTC’s Coordinating Center’s central activities are a supplement and in complement to local site endeavors via continued and robust community and site engagement. Thus far, these strategies have resulted in increased engagement and screening in preclinical AD trials across diverse communities.

**Conclusion:**

As we actively implement and expand these strategies, discussions evolve around early successes, lessons learned, and new and persisting challenges. We discuss centrally coordinated community screening pilot studies that are currently under evaluation and will help identify eligible individuals based on biomarker assessments before site referral. Additionally, we explore opportunities to further improve inclusivity by expanding into other dimensions of diversity through the study of social determinants of health that may influence cognitive health in groups that are disproportionately biomarker‐ineligible for AD trials.

